# Supplementary material for: Simultaneous Analysis of 53 Pesticides in Safflower (Carthamus tinctorius L.) by Using LC–MS/MS Coupled with a Modified QuEChERS Technique
Source: Toxics. 2023 Jun 16;11(6):537. doi: 10.3390/toxics11060537 (PMC10300687; doi:10.3390/toxics11060537)
Supplement: Supplementary file 1 [file toxics-11-00537-s001.zip › toxics-2411135-supplementary.pdf]

*Supplementary Materials*

# Simultaneous Analysis of 53 Pesticides in Safflower (*Carthamus tinctorius* L.) by using LC–MS/MS Coupled with a Modified QuEChERS Technique

**Wei Song [1,2,*,†], Chuanyi Peng [2,3,†], Yuxin Liu [1,2], Fang Han [1,2], Haitao Zhu [1,2], Dianbing Zhou [1,2], Yu Wang [1,2], Lijun Chen [1,2], Xiaodi Meng [1,2] and Ruyan Hou [2,3]**

**Table S1.** MS parameters of 53 pesticides.

| Pesticide | Qualitative Ion Pair | Quantitative Ion Pair | FP(V) | CE (eV) | RT |
|---|---|---|---|---|---|
| Carbendazim | 192.1/160 | 192.1/132 | 118 | 13,25 | 2.8 |
| Thiabendazole | 202/175 | 202/131 | 120 | 30,30 | 3.0 |
| Thiophanate-Methyl | 343/311 | 343/151 | 120 | 10,20 | 5.6 |
| Simetryn | 214/144 | 214/124 | 120 | 20,20 | 5.2 |
| Terbumeton | 226.2/170 | 226.2/114 | 120 | 15,20 | 5.7 |
| Rabenzazole | 213.1/172 | 213.1/118 | 122 | 21,37 | 6.5 |
| Carbofuran | 222/165.1 | 222/123 | 92 | 8,16 | 6.1 |
| Carbaryl | 202/145 | 202/127 | 80 | 5,10 | 6.4 |
| Thiofanox | 241/184 | 241/57.1 | 100 | 5,20 | 6.6 |
| Cycluron | 199.1/89.1 | 199.1/72.2 | 106 | 13,21 | 6.6 |
| Methoprotryne | 272.2/198.1 | 272.2/170.1 | 140 | 20,30 | 6.6 |
| Ametryn | 228/186 | 228/96 | 120 | 20,25 | 6.7 |
| Norflurazon | 304/284 | 304/160 | 140 | 25,35 | 7.2 |
| Pyrimethanil | 200.2/168.1 | 200.2/107 | 41 | 40,34 | 7.6 |
| Chlorantraniliprole | 484/453 | 484/286 | 102 | 13,10 | 8.4 |
| Prometryn | 242.2/200.2 | 242.2/158.1 | 120 | 20,20 | 8.7 |
| Flonicamid | 230/203.1 | 230/174.1 | 120 | 13,17 | 9.0 |
| Mefenacet | 299/148 | 299/120 | 100 | 15,25 | 11.1 |
| Cyprodinil | 226.1/118 | 226.1/93.1 | 152 | 49,37 | 11.9 |
| Mandipropamid | 412.1/328 | 421.1/125 | 100 | 10,40 | 12.0 |
| Bupirimate | 317/272 | 317/166 | 120 | 20,25 | 12.6 |
| Metconazole | 320/125 | 320/70 | 110 | 40,25 | 12.8 |
| Isazofos | 314.1/162.1 | 314.1/120 | 120 | 15,20 | 12.9 |
| Rotenone | 395.1/213 | 395.1/192 | 130 | 20,25 | 13.4 |
| Triflumuron | 359/156 | 359/139 | 120 | 15,25 | 14.6 |
| Difenoconazole | 406/337 | 406/251 | 160 | 15,20 | 14.9 |
| Diazinon | 305.1/169 | 305.1/153 | 140 | 20,20 | 15.0 |
| Triflumizole | 346/278 | 346/73 | 80 | 5,10 | 15.2 |
| Myclobutanil | 289.2/125 | 289.2/70 | 60 | 35,15 | 10.9 |
| 3-Hydroxycarbofuran | 238/163 | 238/181 | 69 | 8,4 | 3.6 |
| Aldicarb | 208.1/116 | 208.1/89.1 | 62 | 5,13 | 2.4 |
| aldicarb sulfone | 223/148 | 223/86 | 111 | 4,12 | 2.7 |
| aldicarb sulfoxide | 207/132 | 207/89 | 77 | 4,10 | 2.3 |
| Fenobucarb | 208.2/152.1 | 208.2/95 | 80 | 5,10 | 9.0 |
| Isoprocarb | 194.1/137 | 194.1/95 | 75 | 5,10 | 7.4 |
| Methomyl | 163.2/106 | 163.2/42.1 | 50 | 8,20 | 3.1 |
| Metolcarb | 166.1/109 | 166.1/91 | 80 | 5,10 | 15.8 |
| Propoxur | 210/168 | 210/111 | 80 | 5,10 | 5.7 |
| Imidacloprid | 256.1/209 | 256.1/175.1 | 106 | 13,13 | 3.8 |

| | | | | | |
|---|---|---|---|---|---|
| Thiamethoxam | 292/181.03 | 292/108 | 80 | 20,29 | 3.2 |
| Clothianidin | 250.2/169.1 | 250.2/132 | 80 | 10,15 | 3.6 |
| Thiacloprid | 253/186 | 253/126 | 120 | 10,15 | 4.6 |
| Acetamiprid | 223.1/126 | 223.1/90 | 102 | 21,13 | 4.0 |
| Chlorpyrifos | 350/198 | 350/97.1 | 100 | 15,30 | 17.8 |
| Fenthion | 279/247 | 279/169 | 100 | 10,15 | 14.8 |
| Coumaphos | 363/307 | 363/227 | 120 | 15,20 | 15.7 |
| Propachlor | 212.1/170 | 212.1/152 | 80 | 10,15 | 4.4 |
| Atrazine | 216/174 | 216/132 | 100 | 15,20 | 6.5 |
| Acetochlor | 270.1/224 | 270.1/148 | 90 | 5,20 | 12.7 |
| Metolachlor | 284.2/252.2 | 284.2/176.2 | 90 | 8,24 | 12.4 |
| Dimethenamid | 276.1/244 | 276.1/168 | 120 | 10,15 | 9.7 |
| Alachlor | 270/238 | 270/162 | 80 | 10,20 | 12.6 |
| Butachlor | 312.1/238 | 312.1/162 | 80 | 10,20 | 17.5 |

**Table S2.** Standard quantitative curves of 53 pesticides.

| Pesticide | Linear Regression Equation | Correlation Coefficient ($R^2$) |
|---|---|---|
| Carbendazim | y=2272.1x+1086.9 | 0.9995 |
| Thiabendazole | y=1802.3x+1718.3 | 0.9997 |
| Thiophanate-Methyl | y=926.4x+285.6 | 0.9983 |
| Simetryn | y=1741.5x+51.7 | 1.0000 |
| Terbumeton | y=1453.4x-454.6 | 0.9991 |
| Rabenzazole | y=366.2x+47.2 | 0.9979 |
| Carbofuran | y=1541.8x-126.8 | 0.9999 |
| Carbaryl | y=1761.3x+143.9 | 0.9988 |
| Thiofanox | y=155.5x-13.4 | 0.9993 |
| Cycluron | y=657.3x+43.3 | 0.9991 |
| Methoprotryne | y=1815.8x+84.0 | 0.9985 |
| Ametryn | y=1301.5x+67.8 | 0.9998 |
| Norflurazon | y=863.2x+81.5 | 0.9992 |
| Pyrimethanil | y=247.3x+147.2 | 0.9999 |
| Chlorantraniliprole | y=593.2x+318.1 | 0.9982 |
| Prometryn | y=2128.5x+451.5 | 0.9998 |
| Flonicamid | y=353.7x-245.8 | 0.9986 |
| Mefenacet | y=1317.1x-16.4 | 1.0000 |
| Cyprodinil | y=468.0x+11.7 | 0.9999 |
| Mandipropamid | y=855.9x+160.8 | 0.9989 |
| Bupirimate | y=593.7x+35.2 | 0.9985 |
| Metconazole | y=362.2x+102.8 | 0.9993 |
| Isazofos | y=1912.1x+1245.8 | 0.9983 |
| Rotenone | y=651.5x+223.5 | 0.9994 |
| Triflumuron | y=931.1x+125.6 | 0.9991 |
| Difenoconazole | y=347.5x-36.0 | 0.9988 |
| Diazinon | y=820.6x-371.0 | 0.9995 |
| Triflumizole | y=2049.5x+848.2 | 0.9998 |
| Myclobutanil | y=778.2x+363.8 | 0.9994 |
| 3-Hydroxycarbofuran | y=514.2x-260.8 | 0.9989 |
| Aldicarb | y=653.4x+405.9 | 0.9991 |
| aldicarb sulfone | y=498.6x-265.6 | 0.9978 |
| aldicarb sulfoxide | y=641.7x+421.6 | 0.9981 |
| Fenobucarb | y=661.3x+55.0 | 0.9998 |
| Isoprocarb | y=2203.1x+38.5 | 0.9991 |

| | | |
|---|---|---|
| Methomyl | y=1398.4x+148.9 | 0.9987 |
| Metolcarb | y=1408.8x-44.4 | 0.9990 |
| Propoxur | y=2328.1x+55.1 | 0.9999 |
| Imidacloprid | y=364.6x+821.4 | 0.9976 |
| Thiamethoxam | y=593.3x+120.7 | 0.9992 |
| Clothianidin | y=364.8x+184.1 | 0.9985 |
| Thiacloprid | y=1136.4x+779.2 | 0.9979 |
| Acetamiprid | y=852.6x-41.8 | 0.9982 |
| Chlorpyrifos | y=214.3x-79.8 | 0.9989 |
| Fenthion | y=331.6x-239.2 | 0.9981 |
| Coumaphos | y=304.3x+108.1 | 0.9983 |
| Propachlor | y=1482.2x+150.3 | 0.9998 |
| Atrazine | y=628.8x+108.6 | 0.9996 |
| Acetochlor | y=554.9x-362.3 | 0.9992 |
| Metolachlor | y=1832.2x+13.8 | 0.9989 |
| Dimethenamid | y=1517.2x+98.8 | 0.9996 |
| Alachlor | y=415.4x-111.2 | 0.9991 |
| Butachlor | y=303.2x-15.6 | 0.9993 |
